# Health services research in colorectal cancer: a quasi-experimental interventional pilot study on in- and outpatient oncology

**DOI:** 10.1007/s00432-020-03454-w

**Published:** 2020-12-29

**Authors:** Margarete Reiter, Michael Gerken, Patricia Lindberg-Scharf, Alois Fuerst, Gudrun Liebig-Hörl, Olaf Ortmann, Ingeborg Eberl, Sabine Bartholomeyczik

**Affiliations:** 1 Diplom-Kauffrau, Faculty of Health, School of Nursing Science, PhD Student at the Witten/Herdecke University, Stockumer Straße 12, 58453 Witten, Germany; 2 Caritas-Hospital Sankt Josef, Landshuter Straße 65, 93053 Regensburg, Germany; 3 Tumor Center Regensburg—Institute of Quality Management and Health Services Research of the University of Regensburg, Coordination, Epidemiology, Am Biopark 9, 93053 Regensburg, Germany; 4 Tumor Center Regensburg—Institute of Quality Management and Health Services Research of the University of Regensburg, Section Quality of Life, Am Biopark 9, 93053 Regensburg, Germany; 5 Director of the Department of General and Visceral Surgery, Caritas-Hospital Sankt Josef, Landshuter Straße 65, 93053 Regensburg, Germany; 6 Medical Coordinator of Colon Cancer Center, Caritas-Hospital Sankt Josef, Landshuter Straße 65, 93053 Regensburg, Germany; 7 Director of the Department of Gynecology and Obstetrics, University Medical Center Regensburg, Caritas-Hospital Sankt Josef, Landshuter Straße 65, 93053 Regensburg, Germany; 8 Faculty of Social Work, Catholic University of Eichstätt-Ingolstadt, Kapuzinergasse 2, 85072 Eichstätt, Germany; 9 Faculty of Health, School of Nursing Science, Witten/Herdecke University, Stockumer Straße 12, 58453 Witten, Germany

**Keywords:** Health services research, Colorectal cancer, Drug-related side effects and adverse reactions, Oncology nurse counseling, Nutritional counseling

## Abstract

**Introduction:**

Due to frequent treatment side effects and weight loss, colorectal cancer patients require oncologic care and nutritional counseling both during and after hospitalization. The current study evaluated differences in discharge and side effects management and nutritional behavior between colorectal cancer patients of a control group without systematic counseling and of an intervention group with access to structured in- and outpatient oncology nurse and nutritional counseling.

**Methods:**

The presented explorative, quantitative, single-center, interventional pilot study is a health services research project with a quasi-experimental design. Using a self-designed standardized questionnaire, data were collected from the control group (*n* = 75) before and from the intervention group (*n* = 114) after the introduction of in- and outpatient oncology nurse and structured systematic nutritional counseling. The in- and outpatient counseling services were developed and evaluated in the form of a structured nurse-led counseling concept.

**Results:**

Intervention group patients profited significantly from inpatient oncology nurse counseling in seven different areas of discharge management. No differences were observed concerning patient-reported general and gastrointestinal side effects except for xerostomia and dysphagia, but of the patients participating in both in- and outpatient oncology nurse counseling, 90.0% were better able to cope with general side effects of treatment. Patients with in- and outpatient structured systematic nutritional counseling more frequently received nutritional information (*p* = 0.001), were better at gauging food intolerances (*p* = 0.023), and followed the dietician's advice in cases of gastrointestinal side effects significantly more often (*p* = 0.003) than control patients. Counselor-reported outcomes concerning gastrointestinal side effects showed improvement in most of the patients taking part in systematic in- and outpatient nutritional counseling, except for weight loss in 4 patients.

**Conclusion:**

In- and outpatient counseling in discharge and side effects management and nutrition improve the outcomes of colorectal cancer patients. Outpatient counseling should be further developed and evaluated in future studies.

## Introduction

With almost 59,000 new cases each year, colorectal cancer is the second most common malignant disease in Germany (Robert-Koch-Institut 2017). Patients frequently lose 5–10% of their body weight within 2–3 months, and suffer from various adverse physical and psychological effects of (neo)adjuvant radio-/chemotherapy such as appetite loss, nausea and vomiting, abdominal pain, diarrhea, constipation, flatulence, mucositis, dysosmia, dysgeusia, difficulty chewing, xerostomia, hand-foot syndrome, sleep disorders, fatigue syndrome, and anxiety and depression (Kreitler 2019; Adlard et al. 2016; Van Vulpen et al. 2016; Middleton 2014; Anderson et al. 2013; Ravasco et al. 2012; Hartinger et al. 2012).

Colorectal cancer patients require professional help to develop self-management strategies to deal with anxiety and insecurity (Appleton et al. 2018) and disease-/treatment-related symptoms (Anderson et al. 2013). They require qualified counseling to stabilize their body weight through sufficient intake of energy and protein (Arends et al. 2017) and also to guide physical activity (Jensen et al. 2014), promote general wellbeing (Appleton et al. 2018), and cope with their disease and its treatment in everyday life (Stuhlfauth et al. 2018). High-quality cancer care should thus incorporate such measures until completion of treatment (Kreitler 2019).

Several studies have shown that colorectal cancer patients have a requirement for nurse counseling regarding the management of symptoms and side effects (Tung et al. 2016; Shun et al. 2014; Jorgensen et al. 2012). In addition, there is currently a lack of counseling on activities of everyday living as well as on psychological and psychosocial needs, and there are problems in communication between healthcare professionals and cancer patients (Steven et al. 2019; Sakamoto et al. 2017; Harrison et al. 2009). Furthermore, a significant proportion of under-/malnourished cancer patients still do not receive adequate nutritional support (Caccialanza et al. 2017; Hébuterne et al. 2014). In Germany, deficits or even discontinuations in care can arise during the transition from the inpatient to the outpatient setting, due to different financing systems (Schlüchtermann 2020). Due to the rising incidence of cancer, outpatient support is becoming particularly important—an aspect that is barely researched and a need which is currently not well met in Germany (Stiel et al. 2009). Therefore, appropriate interventions offered by nursing and medical staff are needed to reduce these deficits in patient care. Based on the considerations outlined above, a complex intervention was developed with the aim of satisfying these unmet demands in patient care. The objective of the current health services research study was to evaluate the concept of optimized nursing and nutritional care of colorectal cancer patients in in- and outpatient settings.

The following research question was addressed: “What differences are present among a collective of colorectal cancer patients divided into a control group and an intervention group with two subgroups, one of which used inpatient and outpatient support services for oncology nurse and nutritional counseling, and the other of which used inpatient counseling services only?”.

The hypothesis was that the implemented intervention with in- and optional outpatient oncology nurse counseling and systematic nutritional counseling improves discharge and side effects management as well as nutritional behavior in colorectal cancer patients.

## Materials and methods

### Design and objective

The current explorative, quantitative, single-center, interventional pilot study with a quasi-experimental design aimed to evaluate differences in discharge and side effects management as well as nutritional behavior between (1) colorectal cancer patients who received inpatient and optional outpatient oncology nurse counseling as well as systematic and optional outpatient nutritional counseling (intervention group) and (2) colorectal cancer patients who received routine care with no nurse counseling and only unsystematic nutritional counseling (control group). The intervention group was further divided into two subgroups: one subgroup comprising patients who received inpatient oncology nurse and systematic nutritional counseling only and another subgroup comprising patients who used the offer of in- and outpatient oncology nurse and systematic nutritional counseling.

### Participants and setting

The study was performed in two general surgery wards of a colorectal cancer center in the Caritas Sankt Josef Hospital in Regensburg, Bavaria, Germany. Patients were treated according to the German S3 guideline “Colorectal Cancer” (Leitlinienprogramm Onkologie AWMF 2014). Exclusion criteria were age < 18 years, dementia, palliative intent, and language and communication problems (Zaner 2015). Participants comprised all primary colorectal cancer patients consenting to participate, assigned to the following groups depending on treatment date: January 2014–April 2015: control group; May 2015–August 2016: intervention group. The control group was surveyed first. Thereafter, a complex intervention comprising structured in- and outpatient oncology nurse and nutritional counseling was implemented. Subsequently, the intervention group was surveyed.

### Complex intervention

The structured counseling concept and complex intervention developed by the authors encompassed two components addressing treatment side effects (oncology nurse counseling) and nutrition (systematic nutritional counseling) and were tailored to different patient profiles. Until completion of treatment, colorectal cancer patients were offered easily accessible in- and outpatient counseling. The specific components of the intervention are described according to the TIDieR checklist (Hoffmann et al. 2014).

### Oncology nurse counseling to promote patient self-management

The intervention was based on the two concepts of health promotion from Antonovsky’s salutogenesis (1997) and Collins and Rochfort’s empowerment (2016), with the promotion of patients' own skills and abilities for autonomous decision-making and independent action. By means of targeted knowledge transfer and practical guidance, patients should be able to better deal with symptoms and general treatment side effects to avoid care deficits.

During inpatient treatment, oncology nurses advised colorectal cancer patients in the intervention group regarding preparation for discharge. Patients received information and instructions on behavior after surgery, physical activity, defecation, and, if necessary, on adjuvant treatment. Furthermore, they received a booklet with specific recommendations for side effect management during adjuvant chemotherapy. Four weeks after discharge, oncology nurses contacted patients at home via telephone. If patients required to support or advice, they received an appointment for outpatient counseling. An additional risk assessment was not performed. Appointments took place on an individual basis, either face-to-face in the hospital or, if preferred by the patient, via telephone. During these sessions, the status of symptoms and side effects was ascertained (Dodd et al. 2001). According to their needs, patients received advice/self-management instructions on mucositis (Dodd and Miaskowski 2000), hand-foot syndrome (Hartinger et al. 2012), fatigue, and sleep disorders (Aapro et al. 2017; Adlard et al. 2016; Van Vulpen et al. 2016), pain (Drury et al. 2017), and coping with everyday life (Stuhlfauth et al. 2018). Furthermore, patients' anxieties and worries were discussed (Appleton et al. 2018; Middleton 2014), and a psycho-oncologist consulted if required (Kreitler 2019). Patients were also instructed in behavioral strategies (Kwekkeboom et al. 2018) and progressive muscle relaxation techniques (Kim et al. 2016) to relax as well as aromatherapy to reduce nausea and vomiting (Zorba and Ozdemir 2018).

Oncology nurse counseling was conducted by two experienced nurses with training in oncologic care and psycho-oncology. Thus, patients had a constant contact person during in- and outpatient care. In preparation for the study, nurses received a one-day training in the oncology nurse counseling intervention from a research group member (MR). During the first 3 months after the implementation of oncology nurse counseling, there were weekly meetings between the nurses and MR to maintain the intervention. There were no changes in the intervention during the study. Counseling was offered in the form of individual counseling.

Patients required an average of 2.8 30-min appointments within the first 6 months after discharge from the hospital until completion of adjuvant chemotherapy. There was no upper limit to the number of consultations.

### Systematic nutritional counseling to promote patient self-management

With systematic in- and outpatient nutritional counseling, patients should be able to reduce gastrointestinal symptoms and side effects of treatment independently and be able to stabilize their weight due to sufficient intake of energy and protein. All patients received a brochure compiled by a clinical dietician with information on nutrition and management of gastrointestinal symptoms and treatment side effects.

Additionally, patients of the intervention group received their first systematic nutritional counseling session from a clinical dietician according to the S3 guideline "Clinical Nutrition in Oncology" (Leitlinienprogramm Onkologie 2015) during their stay in hospital. In cases of metabolic risk or under-/malnutrition (body mass index, BMI: ≤ 18.5 kg/m^2^) according to Nutritional Risk Screening (NRS 2002; Kondrup et al. 2003), patients were transferred to outpatient nutritional counseling. In addition, outpatient counseling services were recommended to patients who needed adjuvant chemotherapy.

During systematic outpatient nutritional counseling, side effects (abdominal pain, nausea, and vomiting, diarrhea, constipation, flatulence, loss of appetite) and weight were assessed.

The systematic counseling took place at the hospital on an individual basis with a clinical dietician according to the 2015 German S3 guideline "Clinical Nutrition in Oncology." Due to the large catchment area of the hospital, consultations were occasionally carried out by telephone. The following areas were addressed: the selection of suitable foods and drinks with concrete portion sizes; methods of food preparation; distribution of total quantities over smaller more frequent meals; enrichment of meals with sources of energy and protein; and oral nutritional supplementation (Lin et al. 2017). Between counseling sessions, patients kept a food and bowel diary, which was used to adapt the specific nutritional recommendations after each session depending on side effects and body weight (Arends et al. 2017).

Two clinical dieticians with at least 3 years of training in nutritional counseling and several years' experience in oncology conducted the in- and outpatient nutritional counseling. After the implementation of the nutritional counseling intervention, there were repeated meetings between the clinical dieticians and MR. Again, counseling was offered on an individual basis. There were no changes in the intervention during the study.

Patients required an average of 2.6 30-min outpatient nutritional counseling sessions within 6 months of completing adjuvant chemotherapy. There was no upper limit to the number of consultations.

### Instruments and data collection

Since validated instruments to evaluate the multimodal intervention were unavailable, a self-designed questionnaire was used. Apart from two open questions, standardized questions with closed-ended answers on a four-step rating scale were used (Gideon 2012): 5 items on sociodemographic characteristics, 19 on discharge management, 17 on treatment side effects, 13 on support offers, and 13 questions on nutrition during treatment; 2 further questions on the use of in- and outpatient oncology nurse and nutritional counseling were provided only to intervention group patients. The questionnaire was evaluated with a classical pretest. With Cronbach's alpha for four sets of questions each comprising 5–13 individual questions ranging from 0.66 to 0.80, the internal consistency of the questionnaire was acceptable (De Smith 2018).

Data were collected about 6 months after surgery. Due to the timeframe of 16 months defined by the hospital for enrollment of each group, we expected to include approximately 150 patients in the control and 150 patients in the intervention group.

The course of outpatient counseling in the intervention group was documented in the hospital information system (HIS) by the oncology nurses and clinical dieticians and data were analyzed.

### Statistical analysis

Data were analyzed using SPSS version 25 (IBM Corp., Armonk, NY, USA). Descriptive statistics employed univariate methods and relative frequencies are reported in tables and figures. Pearson's nonparametric χ^2^ test was used to test the independence of paired categorical variables (De Smith 2018). Significance was set at *p* < 0.05.

Subgroup analyses were performed by subdividing the intervention group into patients who received only inpatient counseling and patients who received both in- and outpatient counseling. Subgroups were compared with control group patients. Correction for multiple testings was not applied.

## Results

A total of 141 questionnaires were sent to control group patients, of which 75 were returned (return rate of 53.2%). A total of 153 questionnaires were sent to intervention group patients, of which 114 were returned (return rate of 74.5%).

Sociodemographic and treatment characteristics of the total sample of 189 patients as well as of the control and intervention arms are shown in Table 1. Only in terms of gender distribution was there a significant difference between the control and intervention groups (*p* = 0.022).

**Table 1:** Comparison of sociodemographic and treatment variables in control and total intervention group

	Control group (*n* = 75)		Total intervention group (*n* = 114)		Total (*n* = 189)		*p*-value
	*n*	%	*n*	%	*n*	%	
Gender							
Female	23	30.7	54	47.4	77	40.7	0.022
Male	52	69.3	60	52.6	112	59.3	
Age (years)							
< 50	7	9.3	8	7.0	15	7.9	0.545
50–59	16	21.3	24	21.1	40	21.2	
60–69	18	24.0	38	33.3	56	29.6	
≥ 70	34	45.3	44	38.6	78	41.3	
Marital status/living arrangement							
Married/living with partner	61	81.3	86	75.4	147	77.8	0.472
Living with children/other relatives	2	2.7	7	6.1	9	4.8	
Living alone	12	16.0	21	18.4	33	17.5	
Treatment							
Op	74	98.7	112	98.2	186	98.4	0.821
Rad/chemo	26	34.7	34	29.8	60	31.7	0.484
Chemo	35	46.7	57	50.0	92	48.7	0.654

*P *values refer to X^2^ tests

*Op* surgery, *rad/chemo* radiochemotherapy, *chemo* chemotherapy

### Oncology nurse counseling

#### Discharge management

Table 2 shows better results for the intervention group compared to the control group in the following aspects of discharge management: more frequent notice of discharge date 2 days in advance (77.2%; *p* = 0.045), more frequent discharge consultation with a physician (85.1%; *p* = 0.017), more opportunities for the discussion of worries and troubles (47.4%; *p* = 0.004), better inclusion of relatives in the course of treatment (51.8; *p* = 0.018), more information on social law issues (61.4%; *p* = 0.015), more frequent transfer to outpatient nutritional counseling (54.4%; *p* < 0.001), and colorectal cancer brochures more frequently distributed to patients (86.0%; *p* < 0.001)

**Table 2:** Hospital discharge procedure in the control and intervention groups

	Control group: no nurse counseling (*n* = 75)		Total intervention group: inpatient and optional outpatient nurse counseling (*n* = 114)		*p*-value
	*n*	%	*n*	%	
Notice of discharge date 2 days in advance	51	68.0	88	77.2	0.045
Discharge consultation with a physician	54	72.0	97	85.1	0.017
Discussion of worries and troubles	22	29.3	54	47.4	0.004
Inclusion of relatives in the course of treatment	32	42.7	59	51.8	0.018
Information on social law issues	29	38.7	70	61.4	0.015
Transfer to outpatient nutritional counseling	18	24.0	62	54.4	< 0.001
Receipt of brochures on colorectal cancer	45	60.0	98	86.0	< 0.001

*P* values refer to X^2^ tests

#### General treatment side effects

The data presented in Table 3 on general treatment side effects relate to the time period between discharge from hospital until 1 month after completion of adjuvant chemotherapy. There were no significant differences between the control and the intervention group in terms of self-reported general treatment side effects.

**Table 3:** General treatment side effects in the control and intervention groups

	Control group: no nurse counseling (*n* = 75)		Total intervention group: inpatient and optional outpatient nurse counseling (*n* = 114)		*p*-value
	*n*	%	*n*	%	
General side effects	48	64.0	78	69.3	0.448
Fever/allergy	6	8.0	5	4.4	0.299
Oral mucositis	7	9.3	22	19.3	0.063
Dizziness	14	18.7	33	28.9	0.110
Hand-foot syndrome	20	26.7	41	36.0	0.181
Breathing difficulties	8	10.7	12	10.5	0.976
Fatigue syndrome	36	48.0	56	49.1	0.880
Sleep disorders	21	28.0	26	22.8	0.419
Anxiety/depression	14	18.7	23	20.2	0.798

*P *values refer to X^2^ tests

#### Subgroup analysis for general treatment side effects

Even when compared between three study groups, there are no significant differences in the general side effects of treatment (Table 4).

**Table 4:** Subgroup analysis of general treatment side effects in the three study groups with different oncology nurse counseling

	Control group: no nurse counseling (*n* = 75)		Intervention group: inpatient nurse counseling only (*n* = 40)		Intervention group: in- and outpatient nurse counseling (*n* = 74)		*p-*value
	*n*	%	*n*	%	*n*	%	
General side effects	48	64.0	26	65.0	52	70.3	0.697
Fever/allergy	6	8.0	1	2.5	4	5.4	0.478
Oral mucositis	7	9.3	8	20.0	14	18.9	0.175
Dizziness	14	18.7	8	20.0	25	33.8	0.074
Hand-foot syndrome	20	26.7	13	32.5	28	37.8	0.345
Breathing difficulties	8	10.7	5	12.5	7	9.5	0.880
Fatigue syndrome	36	48.0	22	55.0	34	45.9	0.646
Sleep disorders	21	28.0	9	22.5	17	23.0	0.720
Anxiety/depression	14	18.7	6	15.0	17	23.0	0.564

*P *values refer to X^2^ tests

#### Patient-reported outcomes

**Fig. 1:**
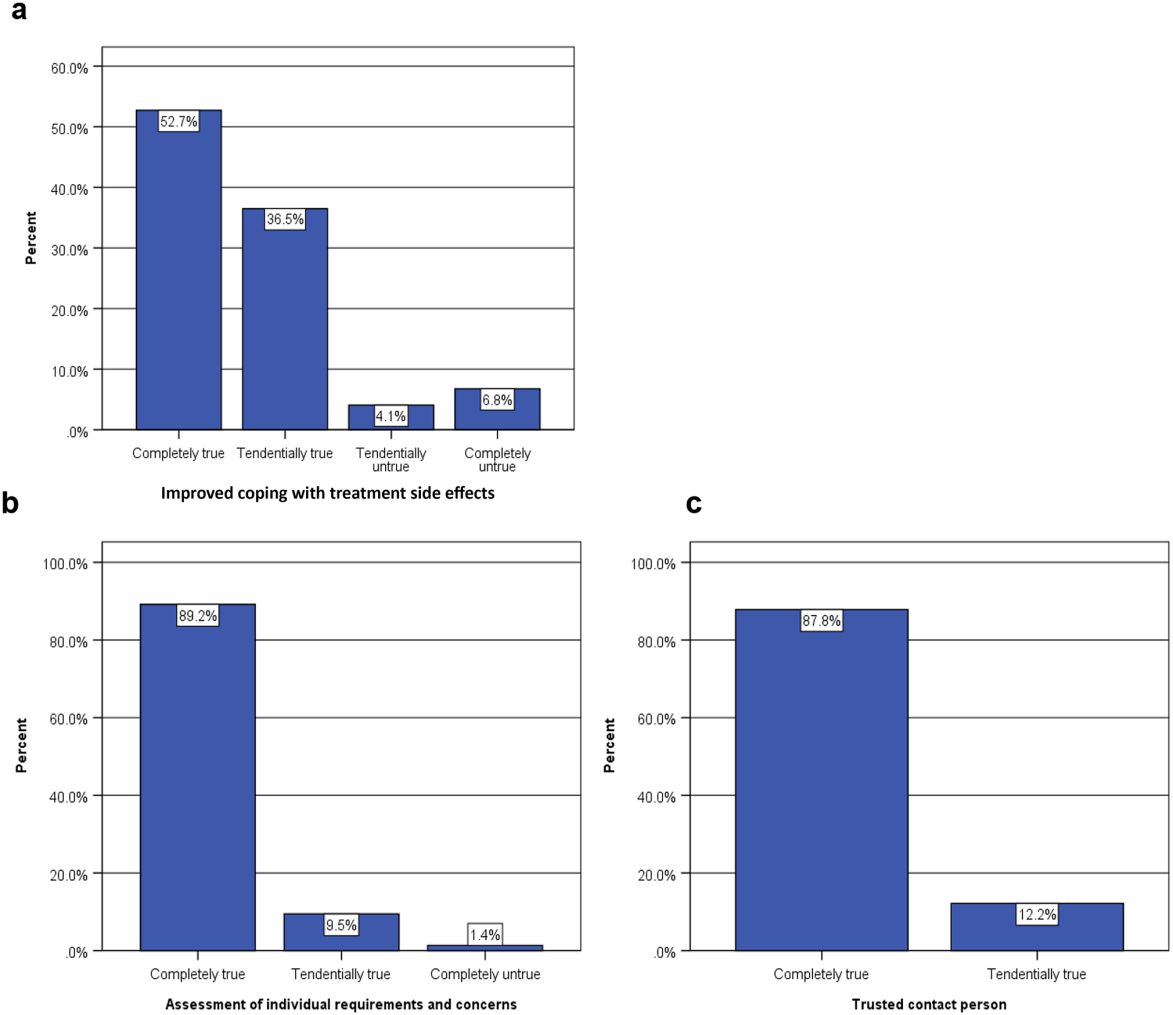
Results of oncology nurse counseling in the intervention group with in- and outpatient counseling (*n* = 74)

Of the 74 patients in the intervention group with in- and outpatient oncology nurse counseling, 66 (89.2%) reported being more able to cope with adverse effects of adjuvant chemotherapy (Fig. 1a). Furthermore, 73 (98.7%) patients were better able to assess their own requirements and concerns (Fig. 1b) and all 74 (100.0%) reported having had a trusted contact person until 1 month after the completion of adjuvant chemotherapy (Fig. 1c).

#### Counselor-reported outcomes (data from the HIS)

In Table 5, six relevant general side effects affecting the 74 patients participating in both in- and outpatient oncology nurse counseling are presented. Moreover, the specific interventions applied to combat these side effects and the outcomes thereof up until 1 month after completion of adjuvant chemotherapy are described.

**Table 5:** Relevant general treatment side effects in patients of the intervention group with in- and outpatient nurse counseling (*n* = 74)

General treatment side effects	*n* (%)	Nursing interventions	Outcomes until 1 month after the end of treatment
Fatigue	34 (45.9)	Progressive muscle relaxation Endurance training targets	Improvement in 28 (82.4%) patients
Sleep disorders	17 (23.0)	Behavioral strategies Progressive muscle relaxation	Improvement in 11 (64.7%) patients
Hand-foot syndrome	28 (37.8)	Cold water baths Oil blends and cremes	Improvement in 21 (75.0%) patients
Anxiety/depression	17 (23.0)	Consultation with nurse Consultation with psycho-oncologist	Improvement in 17 (100.0%) patients
Oral mucositis	14 (18.9)	Self-care instructions for antiseptic/analgesic substances	Improvement in 14 (100.0%) patients
Dizziness and balance disorders	25 (33.8)	Coordination and balance exercises	Improvement in 15 (60.0%) patients

### Nutritional counseling

#### Gastrointestinal side effects

The data on gastrointestinal (GI) treatment side effects relate to the time period between discharge from hospital until 1 month after completion of adjuvant chemotherapy. In the data comparison between the control and intervention groups (Table 6), intervention group patients reported the two GI side effects xerostomia (40.4%; *p* = 0.20) and dysphagia (15.8%; *p* = 0.012) significantly more often.

**Table 6:** Gastrointestinal side effects in the control and intervention groups

	Control group: unsystematic nutritional counseling (*n* = 75)		Total intervention group: systematic inpatient and optional outpatient nutritional counseling (*n* = 114)		*p*-value
	*n*	%	*n*	%	
GI side effects	44	58.7	72	63.2	0.789
Weight loss	26	34.7	49	43.0	0.253
Abdominal pain	15	20.0	17	14.9	0.362
Food intolerances	15	20.0	35	30.7	0.103
Nausea/vomiting	17	22.7	27	23.7	0.871
Diarrhea	27	36.0	53	46.5	0.153
Loss of appetite	19	25.3	37	32.5	0.294
Xerostomia	18	24.0	46	40.4	0.020
Dysphagia	3	4.0	18	15.8	0.012

*P* values refer to X^2^ tests

#### Subgroup analysis for gastrointestinal side effects

Of the 114 patients in the total intervention group, 62 (54.4%) received inpatient systematic nutritional counseling only and 52 (45.6%) participated in- and outpatient counseling.

Patients of the intervention group with in- and outpatient systematic nutritional counseling had significantly more food intolerances (40.4%) than patients in the intervention group with inpatient nutritional counseling only (22.6%) or control patients (20.0%; *p* = 0.026). Furthermore, patients of the intervention group with inpatient systematic nutritional counseling reported significantly more chewing difficulties and dysphagia (17.7%; *p* = 0.032; Table 7).

**Table 7:** Subgroup analysis of gastrointestinal side effects in the three study groups with different nutritional counseling

	Control group: unsystematic nutritional counseling (*n* = 75)		Intervention group: inpatient systematic nutritional counseling only (*n* = 62)		Intervention group: in- and outpatient systematic nutritional counseling (*n* = 52)		*p*-value
	*n*	%	*n*	%	*n*	%	
GI side effects	44	58.7	39	62.9	33	63.5	0.823
Weight loss	26	34.7	25	40.3	24	46.2	0.426
Abdominal pain	15	20.0	9	14.5	8	15.4	0.655
Food intolerances	15	20.0	14	22.6	21	40.4	0.026
Nausea/vomiting	17	22.7	13	21.0	14	26.9	0.745
Diarrhea	27	36.0	27	43.5	26	50.0	0.283
Loss of appetite	19	25.3	17	27.4	20	38.5	0.252
Xerostomia	18	24.0	25	40.3	21	40.4	0.067
Dysphagia	3	4.0	11	17.7	7	13.5	0.032

*GI* gastrointestinal

*P* values refer to X^2^ tests

#### Patient-reported outcomes

Table 8 shows significant differences concerning nutritional advice between the control and intervention groups. Intervention group patients significantly more often reported that they had received extensive information via nutritional counseling (73.6%; *p* = 0.043) and adequate information on nutrition in colorectal cancer (86.0%; *p* < 0.001). Furthermore, they felt able to gauge intolerances or complaints more frequently (68.4%; *p* = 0.012) and followed the recommendations of the nutrition counselor more often (73.7%; *p* = 0.001).

**Table 8:** Results of nutritional counseling in the control and intervention groups

	Control group: unsystematic nutritional counseling (*n* = 75)		Total intervention group: systematic nutritional counseling and optional outpatient nutritional counseling (*n* = 114)		*p*-value
	*n*	%	*n*	%	
Extensive information received via nutritional counseling	40	53.3	84	73.6	0.043
Adequate information on nutrition in colorectal cancer patients	40	53.3	98	86.0	< 0.001
Better gauging of intolerances/complaints	35	46.7	78	68.4	0.012
Followed recommendations of the nutritional counselor in case of adverse events	36	48.0	84	73.7	0.001

*p*-values refer to X^2^ tests

#### Subgroup analysis for patient-reported outcomes

**Fig. 2:**
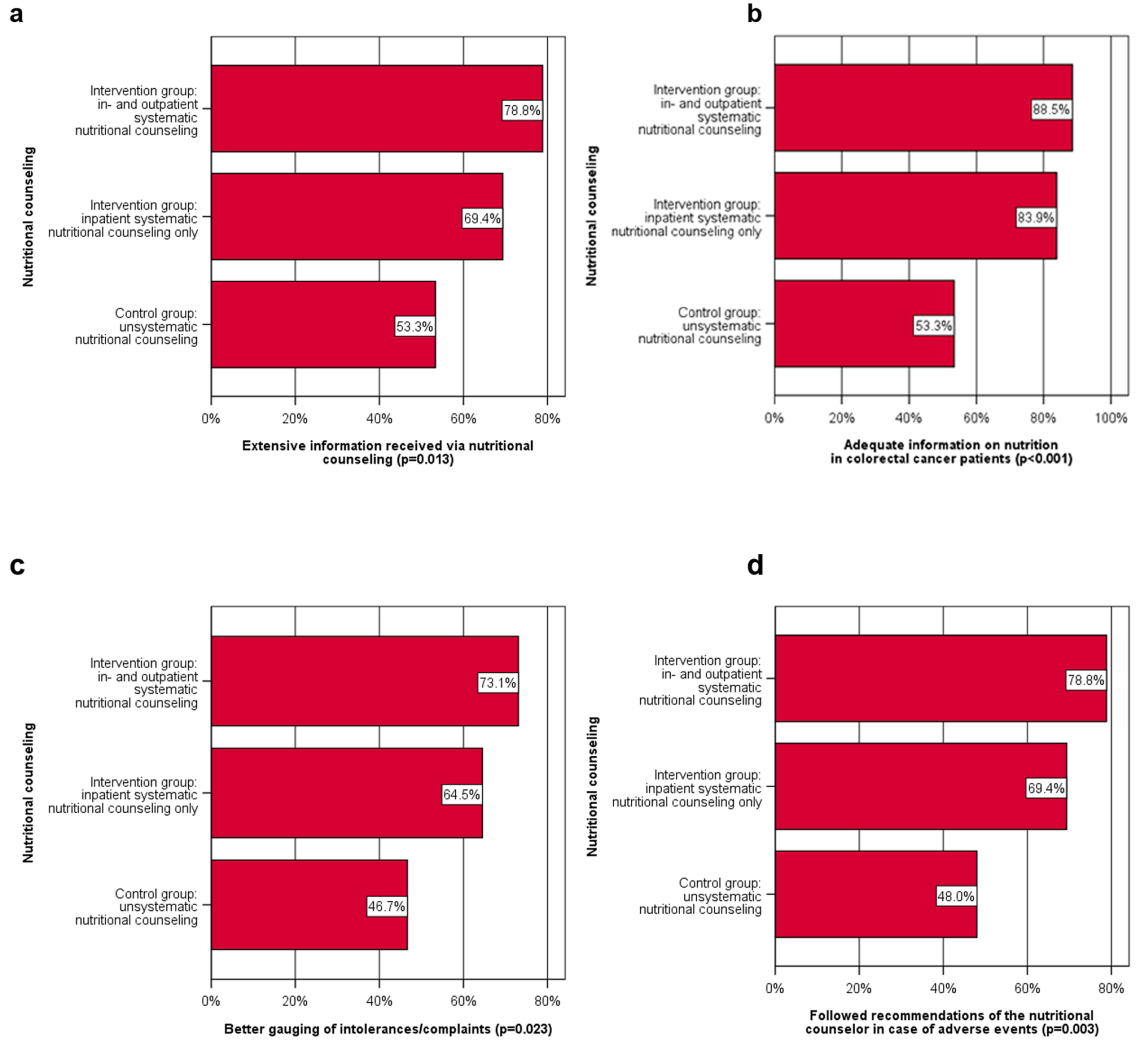
Subgroup analysis of results of in- and outpatient nutritional counseling in the three study groups, *p*-values refer to X^2^ tests

According to the reports of patients of both intervention groups, they received information and concrete instructions regarding the return to solid food and nutrition significantly more often during their stay in hospital (*p* = 0.013; Fig. 2a). Additionally, they more often received satisfactory information on colorectal cancer (*p* < 0.001; Fig. 2b). Moreover, patients of the intervention groups more often reported being better able to gauge food intolerances and GI side effects after in- and outpatient nutritional counseling (*p* = 0.023; Fig. 2c), and followed the counselor`s advice (*p* = 0.003; Fig. 2d).

#### Counselor-reported outcomes (data from the HIS)

In Table 9, six relevant GI side effects affecting the 52 patients participating in both in- and outpatient systematic nutritional counseling are presented. Moreover, the specific interventions applied to combat these GI side effects and the outcomes thereof up until 1 month after completion of adjuvant chemotherapy are described.

**Table 9:** Relevant gastrointestinal side effects in the intervention group with in- and outpatient systematic nutritional counseling (*n* = 52)

Side effect	*n* (%)	Interventions	Outcomes until 1 month after the end of treatment
Weight loss	24 (46.2)	Protein-rich/high-calory diet Dietary supplements	14 (58.3%) weight gain 6 (25.0%) weight stabilization 4 (16.7%) further weight loss
Diarrhea	26 (50.0)	Special diet Well-tolerated foods	Improvements in 21 (80.8%) patients
Food intolerances	21 (40.4)	Easily digestible foods/drinks Food preparation instructions Several small meals a day	Improvements in 19 (90.5%) patients
Loss of appetite	20 (38.5)	Several small meals a day Pepsin wine Eat in company	Improvement in 11 (55.0%) patients
Xerostomia	21 (40.4)	Oral rinses Cold drinks Boiled sweets	Improvement in 21 (100.0%) patients
Nausea/vomiting	14 (26.9)	Aromatherapy Antiemetics	Improvement in 14 (100.0%) patients

## Discussion

Structured outpatient oncology nurse and nutritional interventions for colorectal cancer patients are rare in Germany. The presented study demonstrates that the development of a structured in- and outpatient counseling concept has some positive effects on discharge and side effects management as well as on patients' nutritional behavior. The concept helps to reduce the gap in cancer care. This can only be achieved by interdisciplinary cooperation between treating physicians, oncology nurses, and clinical dietitians.

The age distribution in the current study corresponded to that of a normal colorectal cancer collective in Germany according to the Robert-Koch-Institut (2017). The study groups were imbalanced in terms of gender, an effect which likely resulted from the small size of the study groups. Men develop rectal carcinoma significantly more often, which may explain the gender distribution (Robert-Koch-Institut 2017; Marks 2017).

Furthermore, most patients in the current study lived in a familial environment. Previous studies have shown social support and family solidarity to reduce patients' psycho-emotional stress, promote their wellbeing, and positively influence coping with the disease and its treatment (Stuhlfauth et al. 2018; Corner et al. 2013; Usta 2012).

In contrast to the control group, patients of the intervention group profited significantly from inpatient oncology nurse counseling in seven areas of discharge management. Similar to the findings of Stuhlfauth et al. (2018) and Waring et al. (2014), these patients received more behavioral information and instructions, which eased the transition from hospital to their home environment and gave them more security in everyday life. Effective coordination of this key process and intensive provision of information by physicians and oncology nurses also reduced stress and anxiety in patients and relatives in the study by Carroll and Dowling (2013). In their review, Nosbusch et al. (2011) found that nurses are highly qualified to identify barriers and challenges in discharge management. Furthermore, effective discharge planning can help patients to better manage symptoms and side effects at home.

Of the 74 intervention group patients with in- and outpatient oncology nurse counseling, most were better able to cope with adverse effects of adjuvant chemotherapy. It is assumed that analogous to the effects observed by Aapro et al. (2017), Adlard et al. (2016), Van Vulpen et al. (2016), and Jensen et al. (2014), the training in progressive muscle relaxation and moderate endurance training led to improvements in most patients with fatigue and sleep disorders. In their systematic review of patients with colorectal cancer, Bradenbarg et al. (2017) were unable to demonstrate significant improvements in this regard.

After repeated outpatient practical instruction in the use of the PRO-SELF Program from Dodd & Miaskowski (2000), mucositis improved in all affected patients. Due to the study design, it is not possible to infer a direct effect of the PRO-SELF Program on mucositis. Self-management programs contribute significantly to reducing the burden of symptoms and side effects (Howell et al. 2017). However, some systematic reviews indicate that these programs are limited by a lack of practical instructions and appreciation of cultural differences (Hammer et al. 2015; Gao and Yuan 2011).

As in the studies by Appleton et al. (2018), and Middleton (2014), in- and outpatient oncology nurse counseling in the present intervention also focused on the psychological and emotional status of the patients. Specialized oncology nurses play a key role at the interface between medicine and psychology because they often have more personal and continuous contact with the patients compared to other healthcare professionals, and patients are thus frequently more open toward them. Therefore, nurses can detect changes in patients' psychological well-being early (Mehnert and Lordick 2017). The American Society of Clinical Oncology also recommends the support of qualified oncology nurses to reduce psychosocial distress in cancer patients (Andersen et al. 2014). The investigations of Guo et al. (2013) and Booth et al. (2005) found that cancer patients who received psycho-oncological care from specialized oncology nurses showed a reduction in anxiety and depressed mood. In the present study, a psycho-oncologist was consulted in cases of high-stress levels. In a systematic review of 14 randomized controlled trials (RCT) on psychosocial interventions in patients with colorectal cancer, only three studies showed a significant effect on the reduction of anxiety and depression (Mosher et al. 2017). Since there is currently a lack of standardized interventions in psycho-oncological patient care, systematic reviews and meta-analyses show a high degree of study heterogeneity (Jenniches et al. 2020).

Up until now, only a few studies have investigated the effects of posthospitalization nursing interventions on the outcome of colorectal cancer patients (Zhanga et al. 2014; Anderson et al. 2013; Gray et al. 2013; Grant et al. 2011), although several studies have indicated unmet counseling requirements in this patient group (Sakamoto et al. 2017; Tung et al. 2016; Shun et al. 2014; Jorgensen et al. 2012).

Although many national and international recommendations on nutrition in cancer patients have been published (Arends et al. 2017; August et al. 2009), a large proportion of under-/malnourished patients do not receive adequate dietary support (Caccialanza et al. 2017; Hébuterne et al. 2014). This demonstrates that systematic nutritional interventions are not yet fully established in oncologic practice, although under-/malnourishment during cancer treatment is a negative prognostic factor (Ravasco 2019).

Of the 52 intervention group patients who participated in outpatient nutritional counseling during adjuvant chemotherapy, 38% were able to gain or stabilize their weight through a special protein-rich high-calory diet. The RCT from Ravasco et al. (2012) also showed that intensive nutritional counseling of colorectal cancer patients is the most effective means of improving nutritional status during radiotherapy. Lin et al. (2017) and Dobrila-Dintinjana et al. (2013) also demonstrated that individual nutritional counseling and administration of dietary supplements reduced weight loss and improved appetite in colorectal cancer patients, thus stabilizing nutritional status and reducing chemotherapy-induced morbidity.

In the current study, the intensity of nutritional counseling depended on the frequency of GI side effects, which were most frequent in the intervention group with the use of in- and outpatient counseling. Nutritional and behavioral recommendations in outpatient counseling are assumed to have led to the improvement of diarrhea in almost all patients, reduction of nausea in all patients, and increased appetite in half of the patients. Nutritional behavior is the only factor that patients can control for addressing intolerance and other GI side effects during treatment (Ravasco 2019). According to Cotogni et al. (2019), nutritional counseling in tumor patients helps not only to improve body weight but also to reduce the incidence and severity of toxicity during adjuvant chemotherapy or radiotherapy, so that interruptions to treatment can be avoided.

An effective self-management program—supported by clinical dieticians and nurses—promoting patients' well-being and everyday functioning is thus of central importance (Hammer et al. 2015). In their systematic review of patients with GI and lung cancers, Baldwin et al. (2012) were unable to show the positive effects of oral nutritional interventions. Within a heterogeneous landscape of studies, there are only a few RCTs with well-described and comparable nutritional interventions for cancer patients (Cotogni et al. 2019; Solheim et al. 2019). Varying criteria for the assessment of nutritional status and different nutritional patterns in diverse ethnic groups lead to divergent outcomes.

## Limitations

The small sample sizes of study groups are a limitation of the current work, which may have precluded recognition of relevant differences in the analysis. The power and generalizability of the results are thus limited (De Smith 2018). Bias due to social desirability cannot be excluded. The study design with quasi-experimental, non-randomized groups and lack of blinding precludes conclusions on causality. Furthermore, because of the explorative character of the study and multiple testings, the results should be interpreted in an exploratory manner.

## Conclusion and implications for practice

Oncology nurse and nutritional counseling appear to help patients to develop effective self-management strategies to cope with general and GI treatment side effects and psychosocial stress in everyday life. Patients also have a trusted contact person until the end of treatment. Application of salutogenic (Antonovsky 1997) and empowerment (Collins and Rochfort 2016) concepts also renders patients more able to manage their disease and return to normal life after the end of treatment. Furthermore, inpatient oncology nurse counseling appears to significantly improve discharge management.

Further interventional studies on the care requirements of cancer patients are necessary to develop and implement structured in- and particularly outpatient support services targeting symptom and side effects management, nutritional behavior, and psychosocial counseling (Scott et al. 2017). In light of the increasing number of cancer patients, these interventions must be specific, effective, and sustainable. Outpatient counseling should be available to all cancer patients requiring it.

A positive effect of the current study is that the in- and outpatient counseling services have now been extended to all types of solid tumors at the authors' hospital.
